# TEC-miTarget: enhancing microRNA target prediction based on deep learning of ribonucleic acid sequences

**DOI:** 10.1186/s12859-024-05780-z

**Published:** 2024-04-20

**Authors:** Tingpeng Yang, Yu Wang, Yonghong He

**Affiliations:** 1 Peng Cheng Laboratory, Shenzhen, 518055 China; 2 Tsinghua Shenzhen International Graduate School, Shenzhen, 518055 China

**Keywords:** MicroRNAs, miRNA targets, Target prediction, Deep learning, Transformer encoder, Convolutional neural networks

## Abstract

**Background:**

MicroRNAs play a critical role in regulating gene expression by binding to specific target sites within gene transcripts, making the identification of microRNA targets a prominent focus of research. Conventional experimental methods for identifying microRNA targets are both time-consuming and expensive, prompting the development of computational tools for target prediction. However, the existing computational tools exhibit limited performance in meeting the demands of practical applications, highlighting the need to improve the performance of microRNA target prediction models.

**Results:**

In this paper, we utilize the most popular natural language processing and computer vision technologies to propose a novel approach, called TEC-miTarget, for microRNA target prediction based on transformer encoder and convolutional neural networks. TEC-miTarget treats RNA sequences as a natural language and encodes them using a transformer encoder, a widely used encoder in natural language processing. It then combines the representations of a pair of microRNA and its candidate target site sequences into a contact map, which is a three-dimensional array similar to a multi-channel image. Therefore, the contact map's features are extracted using a four-layer convolutional neural network, enabling the prediction of interactions between microRNA and its candidate target sites. We applied a series of comparative experiments to demonstrate that TEC-miTarget significantly improves microRNA target prediction, compared with existing state-of-the-art models. Our approach is the first approach to perform comparisons with other approaches at both sequence and transcript levels. Furthermore, it is the first approach compared with both deep learning-based and seed-match-based methods. We first compared TEC-miTarget’s performance with approaches at the sequence level, and our approach delivers substantial improvements in performance using the same datasets and evaluation metrics. Moreover, we utilized TEC-miTarget to predict microRNA targets in long mRNA sequences, which involves two steps: selecting candidate target site sequences and applying sequence-level predictions. We finally showed that TEC-miTarget outperforms other approaches at the transcript level, including the popular seed match methods widely used in previous years.

**Conclusions:**

We propose a novel approach for predicting microRNA targets at both sequence and transcript levels, and demonstrate that our approach outperforms other methods based on deep learning or seed match. We also provide our approach as an easy-to-use software, TEC-miTarget, at https://github.com/tingpeng17/TEC-miTarget. Our results provide new perspectives for microRNA target prediction.

**Supplementary Information:**

The online version contains supplementary material available at 10.1186/s12859-024-05780-z.

## Background

MicroRNAs (miRNAs) are a class of short non-coding RNAs consisting of approximately 22 ribonucleotides. They serve as crucial regulators in gene expression by binding to specific transcripts of target genes, with binding sites referred to as microRNA target sites (miRNA targets). The principal mechanism through which miRNAs regulate their targets involves the binding of miRNAs to messenger RNAs (mRNAs), which subsequently inhibits the translation process, leading to reduced protein synthesis and ultimately down-regulated gene expression [1]. For instance, MicroRNA-138 and MicroRNA-25 have been observed to down-regulate the expression of mitochondrial calcium uniporter, contributing to the development of pulmonary arterial hypertension cancer phenotype [2]. Similarly, Hypoxia-induced MIR155 has been found to impact an increasing number of human diseases by targeting multiple players in the MTOR pathway [3]. Collectively, miRNAs play a pivotal role in post-translational gene regulation underscoring the importance of elucidating their functional significance, with the identification of miRNA targets serving as a key task.

CLIPL (crosslinking and immunoprecipitation followed by RNA ligation) [4] and CLASH (crosslinking, ligation, and sequencing of hybrids) [5] techniques have been employed for the experimental identification of miRNA targets. However, the considerable cost and time overhead required by these methods greatly reduced their practical applications. Computational tools for predicting miRNA targets have been developed shortly after miRNAs were widely identified in the human genome as alternative approaches.

In the early stages, heuristic methods [6] such as PITA [7], mirSVR [8], miRDB [9], microT [10] and Targetscan [11, 12] were used for miRNA target prediction. These methods usually adopt biological or physicochemical targeting features: the sequence complementarity between a miRNA and its target sites, the sequence conservation across species, the change in Gibbs free energy, and the site accessibility in their secondary structure. Subsequently, the advent of deep learning led to substantial improvements in building computational tools for miRNA target prediction. For instance, DeepMirTar [13], based on the stacked denoised autoencoder (SdAE) [14], utilizes 750 features to characterize miRNAs and their candidate target sites (CTS), incorporating expert features such as seed match type and free energy. Another model, miRAW [15], employs an eight-layer deep artificial neural network [16], with the first five layers dedicated to extracting accessibility energy features and the final three layers focused on prediction. Furthermore, miTAR [17] is a hybrid miRNA target prediction model composed of six layers, incorporating both convolutional neural networks (CNNs) [18] and the bidirectional RNN (BiRNN) [19]. Meanwhile, GraphTar [20] approaches miRNA target prediction as a graph classification problem and employs graph neural networks (GNNs) [21] to execute the prediction task. Additionally, deepTarget [22] employs RNNs [19] and introduces an end-to-end learning framework for miRNA target prediction, and deepTargetPro [23] (referred to as deepTargetPro for convenience, as it is an improved version of deepTarget) utilizes a one-dimensional convolutional neural network [18] and experimental negative data to predict microRNA targets, instead of mock data. Moreover, TargetNet [24] adopts a relaxed CTS selection criteria and integrates the ResNet [25] to capture the miRNA–CTS interactions. However, these methods mainly focus on the seed-match features of a miRNA and the CTSs of a mRNA while neglecting the whole sequences, making their performances limited because the structure, energy, and other information of a molecule are determined by its constituent sequence.

In recent years, deep learning has undergone remarkable advancements, with convolutional neural networks (CNNs) [18], recurrent neural networks (RNNs) [19], Transformer [26], and other neural network architectures exhibiting exceptional performance across diverse domains. CNNs excel at extracting key information from images while eliminating redundancy, making them widely adopted for image feature extraction [27]. RNNs and Transformers are extensively utilized in temporal sequence processing tasks, such as machine translation and speech recognition. Comparatively, Transformers leverage self-attention mechanisms to extract superior features when compared to RNNs. Additionally, the Transformer architecture replaces sequential computation in temporal sequences with parallel computation, resulting in significant improvements in training speed [28].

RNA sequences consist of four distinct ribonucleotides, each characterized by a specific base: adenine (A), guanine (G), cytosine (C), and uracil (U). These ribonucleotides are arranged in a specific order, giving rise to the unique sequence of RNAs. As a result, RNA sequences can be regarded as a form of natural language, and their representations can be obtained through the application of natural language processing methods. Similar to sequence-based natural language representation, which embodies the structural and semantic information of sentences, these sequence-based representations also contain the structure, energy, and conservation information for RNAs. Afterward, the fusion of representations for a given miRNA and its candidate target site (CTS) can be achieved by constructing a three-dimensional array, akin to a multi-channel image. Consequently, techniques derived from computer vision can be employed for processing and analyzing this three-dimensional array.

In this study, we present a novel model, TEC-miTarget, for predicting miRNA targets by leveraging the power of the transformer encoder [26] and convolutional neural networks (CNNs). TEC-miTarget employs a transformer encoder to capture meaningful representations of both miRNA and its candidate target site (CTS) sequences. These representations are then fused to generate a contact map, which is subsequently fed into CNNs for feature extraction. Finally, TEC-miTarget predicts whether the CTS sequence is a miRNA target based on the extracted features. Through a comprehensive series of comparative experiments against state-of-the-art models based on deep learning and seed match, we demonstrate that TEC-miTarget achieves significant performance improvements at both the sequence level and transcript level.

## Methods

### Datasets

We obtained three datasets from the studies of miRAW, DeepMirTar, and deepTargetPro. For convenience, we will refer to these datasets as the miRaw dataset, DeepMirTar dataset, and deepTargetPro dataset, respectively. All positive pairs for the three databases, along with the negative pairs for the miRAW and deepTargetPro datasets, are determined through experimental methods. However, for DeepMirTar, the negative pairs are generated by shuffling the real mature miRNAs. The miRaw dataset and DeepMirTar dataset contain only sequence-level (miRNA-CTS sequences) pairs, while the deepTargetPro dataset also includes transcript-level (miRNA-transcript sequences) pairs. It is important to highlight that we have implemented the dataset partitioning method described in the corresponding works to ensure fair comparisons, and we ensure that there is no duplication of data between the training set and the test sets to ensure the integrity of the evaluation process.

The miRAW dataset consists of two parts. The first part is a segmented dataset consisting of a training set with 40,096 pairs (miRAW training set), a validation set with 10,025 pairs (miRAW validation set), and a test set with 12,532 pairs (miRAW test set). In the segmented dataset, the number of positive pairs is approximately the same as the number of negative pairs. Additionally, there is an independent test set with 929 positive pairs and 890 negative pairs (miRAW independent test set).

The DeepMirTar dataset also has two parts. The segmented dataset contains a training set with 4964 pairs (DeepMirTar training set), a validation set with 1242 pairs (DeepMirTar validation set), and a test set with 1552 pairs (DeepMirTar testing set). Similar to the miRAW dataset, the number of positive pairs is approximately the same as the number of negative pairs in the segmented dataset. Furthermore, there is an independent test set with 48 positive pairs (DeepMirTar independent test set).

The deepTargetPro dataset comprises a sequence-level dataset consisting of 33,142 positive pairs and 32,284 negative pairs (deepTargetPro training set). Additionally, there are ten independent transcript-level test sets available, named deepTargetPro test sets 1–10.

The miRAW dataset and DeepMirTar dataset are utilized to assess the performance of TEC-miTarget at the sequence level. Models with the best performance on the validation sets are then evaluated on the corresponding test sets and independent test sets, following the methodologies described in the respective studies.

The deepTargetPro dataset is employed to assess the performance of TEC-miTarget at the transcript level. For this evaluation, the sequence-level dataset (deepTargetPro training set) is utilized for model training. Subsequently, the performance of the trained model is evaluated using ten independent transcript-level test sets. Specifically, CTSs of a miRNA in a transcript are collected, and then TEC-miTarget predicts whether the miRNA has interactions with the CTSs.

### The architecture of TEC-miTarget

**Fig. 1:**
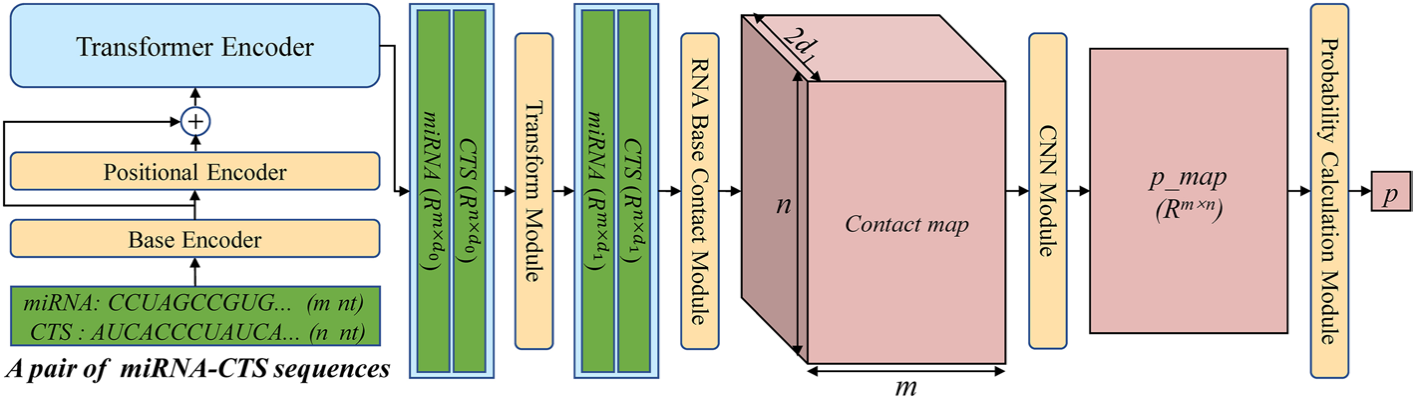
The architecture of TEC-miTarget

TEC-miTarget is a deep learning model consisting of three key components: RNA sequence representation, representation fusion, and interaction prediction. The RNA sequence representation section encodes miRNA and CTS sequences using a base encoder, positional encoder, and transformer encoder, resulting in two groups of representations. In the representation fusion section, these two representations are transformed into appropriate dimensions using a transform module and fused into a contact map using the RNA base contact module. Lastly, the contact map's features are extracted by a CNN module, and the interaction possibility of the miRNA-CTS pair is calculated using the probability calculation module. Refer to Fig. 1 for a graphical representation of the model.

### Base encoder

The base encoder converts an RNA sequence of length $l$ into a tensor of size $l\times {d}_{0}$ (denoted as $X\in {R}^{l\times {d}_{0}}$) using an embedding layer [29]. In this process, the four bases of RNAs (1: A, 2: G, 3: C, 4: U) of RNAs, and zero padding are encoded, requiring a dictionary of embeddings with a size of 5. Furthermore, the embedding vector's dimension is set as ${d}_{0}$ to match the dimension of the transformer encoder.

### Positional encoder

The positional encoder [26] encodes the location information of RNA bases as ${P}_{X}\in {R}^{l\times {d}_{0}}$, and the vector of the i-th base is calculated as follows:

$${P}_{X}\left(i,2j\right)={\text{sin}}(i/{10000}^{2j/{d}_{0}})$$

$${P}_{X}\left(i,2j+1\right)={\text{cos}}(i/{10000}^{2j/{d}_{0}})$$

where $$i=\mathrm{1,2},\cdots ,l$$, $$j=\mathrm{1,2},\cdots ,{d}_{0}/2$$.

### Transformer encoder

The transformer encoder is composed of ${n}_{l}$ encoder layers with ${n}_{h}$ attention heads. The dimension of the transformer encoder is ${d}_{0}$, the feedforward module has a dimension of $2{d}_{0}$, and the dropout parameter is set ${p}_{dropout}$. The transformer encoder takes the sum of $X$ and ${P}_{X}$ as the input, and produces the output (represented as $T$) belonging to ${R}^{l\times {d}_{0}}$.

### The transform module

The transform module consists of a linear layer [30], followed by an activation function $ReLU$ (rectified linear units) [31], and a dropout layer [32]. The output of the transform module is calculated as:

$${T}_{i}^{\mathrm{^{\prime}}}=Dropout\left(ReLU\left({T}_{i}W+b\right),{p}_{dropout}\right), i=1, 2,\cdots , l$$

where $W\in {R}^{{d}_{0}\times {d}_{1}}$ and $b\in {R}^{{d}_{1}}$ represent the learned weights and biases respectively. The $ReLU$ function, also known as the rectified linear unit, is a non-linear activation function defined as $ReLU\left(x\right)={\text{max}}(0,x)$. In addition, the term $Dropout(X,{p}_{dropout})$ refers to the process of randomly setting a fraction ${p}_{dropout} ({p}_{dropout}\in [\mathrm{0,1}])$ of values in $X$ to be zero during training.

### The RNA base contact module

After processing a pair of miRNA-CTS sequences with the base encoder, positional encoder, transformer encoder, and transform module, their representations are obtained as ${T}_{1}{\prime}\in {R}^{m\times {d}_{1}},{T}_{2}{\prime}\in {R}^{n\times {d}_{1}}$, which are also the inputs of the RNA base contact module. To calculate the features ($diff, mul$) for the ${k}_{th}$ dimension, the features between the ${i}_{th}$ base in miRNA and the ${j}_{th}$ base in CTS, namely $dif{f}_{k,i,j}$ and $mu{l}_{k,i,j}$, are computed as:

$$dif{f}_{k,i,j}=\left|{{T}_{1}^{\mathrm{^{\prime}}}}_{i,k}-{{T}_{2}^{\mathrm{^{\prime}}}}_{j,k}\right|$$

$${mul}_{k,i,j}={{T}_{1}^{\mathrm{^{\prime}}}}_{i,k}\times {{T}_{2}^{\mathrm{^{\prime}}}}_{j,k}$$

where $i=1, \cdots , m, j=1, \cdots , n, k=1, \cdots , {d}_{1}$. The $contact\_map$ of the pair of miRNA-CTS is the concatenation of $diff$ and $mul$, resulting in the $contact\_map\in {R}^{2{d}_{1}\times m\times n}$. These two types of features have been utilized in prior research and demonstrated promising performance [33].

### The CNN module

The CNN module primarily comprises four convolutional layers, each accompanied by a batch normalization layer and a nonlinear activation function. The specific parameters for the convolutional layers are provided in Table 1.

**Table 1:** The parameters of four layers in the CNN module

	in_channels	out_channels	kernel_size	Stride	Padding	Activation function
layer 1	$2{d}_{1}$	${d}_{1}$	$ks$	1	$ks//2$	$ReLU(x)$
layer 2	${d}_{1}$	${d}_{1}/2$	$ks$	1	$ks//2$	$ReLU(x)$
layer 3	${d}_{1}/2$	${d}_{1}/4$	$ks$	1	$ks//2$	$ReLU(x)$
layer 4	${d}_{1}/4$	1	$ks$	1	$ks//2$	$\sigma \left(x\right)=\frac{1}{1+{e}^{-x}}$

The expression $ks//2$ denotes the quotient obtained when $ks$ is divided by 2. The base interaction probability map ($p\_map\in {R}^{m\times n}$) is computed after processing the contact map through the CNN module.

### The probability calculation module

In this module, a global pooling operation is applied to $p\_map$, which is calculated as

$$Q=ReLU(p\_map-mean\left(p\_map\right)-\gamma \times var(p\_map))$$

$${p}_{Q}=\frac{{\sum }_{i=1}^{m}{\sum }_{j=1}^{n}{Q}_{i,j}}{{\sum }_{i=1}^{m}{\sum }_{j=1}^{n}sign({Q}_{i,j})+1}$$

$$p=\sigma ({p}_{Q},\eta ,{p}_{0})$$

where

$$\sigma \left({p}_{Q},\eta ,{p}_{0}\right)=\frac{1}{1+{e}^{-\eta \left({p}_{Q}-{p}_{0}\right)}}$$

$$sign\left(x\right)=\left\{\begin{array}{c} 1, x>0\\ 0, x=0\\ -1,x<0\end{array}\right.$$

$mean\left({p}_{map}\right)$, $var\left({p}_{map}\right)$ represent the mean and variance of ${p}_{map}$, respectively. $\gamma$ and $\eta$ are learned parameters, while ${p}_{0}$ is a hyperparameter with a value ranging between 0 and 1.

### Evaluation metrics of the model

Given the labels and predictions of miRNA-CTS sample pairs, the true positive, false positive, true negative, and false negative samples are defined in Table 2:

**Table 2:** The definitions of TP, FP, TN, FN

Predictions	Labels	
	True	False
True	TP	FP
False	FN	TN

Accuracy, sensitivity, specificity, positive predictive value (PPV), negative predictive value (NPV), and F_1_ score are common metrics used for evaluating classification problems, and they are calculated as follows:

$$Accuracy=\frac{TP+TN}{TP+FP+TN+FN}$$

$$Sensitivity=\frac{TP}{TP+FN}$$

$$Specificity=\frac{TN}{TN+FP}$$

$$PPV=\frac{TP}{TP+FP}$$

$$NPV=\frac{TN}{TN+FN}$$

$${F}_{1} score=\frac{2TP}{2TP+FP+FN}$$

AUC and AUPR (area under the precision-recall curve) are additional metrics commonly used in classification tasks. AUC represents the area under the receiver operating characteristic (ROC) curve, whereas AUPR denotes the area under the precision-recall curve.

### Training the model

The base training objective is the binary cross entropy (BCE) loss [34] which is calculated by comparing the predicted probabilities generated by the model with the true binary labels. The model training is conducted using Python 3.8 and PyTorch 1.13.1 on an NVIDIA Tesla V100 with 32 GB of memory. The model weights are initialized with a random seed of 1234. Training is performed for 40 epochs using a batch size of 32 and the Adam optimizer with an initial learning rate of 0.0001. The model's performance is evaluated using the validation set during training, and the best model is determined based on the highest score calculated as follows:

$$score=Accuracy+Sensitivity+Specificity+PPV+NPV+{F}_{1} score$$

Four hyperparameters, namely ${n}_{h}$, ${d}_{1}$, $ks$, and ${p}_{0}$ were determined as 1, 256, 9, and 0.5, respectively, through experimental analysis. The remaining hyperparameters were set as follows, following the convention of classic models: ${d}_{0}=512$, ${n}_{l}=6$, and ${p}_{dropout}=0$.

### Predicting miRNA-RNA interaction at two levels

**Fig. 2:**
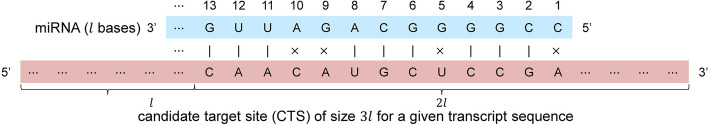
The candidate target site (CTS) of size 3$l$ for a given transcript sequence

The primary function of TEC-miTarget is to predict the interaction between a pair of miRNA-CTS sequences, which is referred to as sequence-level prediction. Using the sequence-level prediction, the transcript-level prediction is subsequently computed as:

1. Given a pair of miRNA-transcript sequences, arrange the miRNA from 5'-end to 3'-end, and the transcript from 3'-end to 5'-end.
2. Calculate the length of the miRNA (represented as $l$).
3. Identify ${n}_{c}$ CTS sequences using a 13-mer-m9 approach: select the Watson–Crick pairings of the first 13 bases in the miRNA as the $seed sequence$, find ${n}_{c}$ short sequences with an edit distance of no more than 4 from the $seed sequence$.
4. Expand the short sequences into CTS sequences using the following method: start from the 5'-ends of each short sequence, expand $l$ bases from 3'-end to 5'-end until reaching the 5'-end, and expand $2l$ bases from 5'-end to 3'-end until reaching the 3'-end.
5. If ${n}_{c}=0$: The pair of miRNA-transcript has no interactions. Else: form ${n}_{c}$ pairs of miRNA-CTS sequences for the transcript, and predict the ${n}_{c}$ pairs of miRNA-CTS sequences using TEC-miTarget and get ${n}_{c}$ predictions, determine the largest prediction value (represented as ${p}_{max}$) among the predictions, and go to the next step.
6. If ${p}_{max}\ge 0.5$: The pair of miRNA-transcript has an interaction. Else: The pair of miRNA-transcript has no interactions.

where the edit distance is defined as the minimum number of insertions, deletions, or substitutions required to transform a sequence into the $seed sequence$. Figure 2 illustrates an example of a candidate target site, in which the symbols $|$ and $\times$ represent whether the base pair satisfies the Waston-Crick condition [35] or not, respectively.

## Results

### Hyperparameter tuning experiments for TEC-miTarget

The TEC-miTarget hyperparameters were optimized using the miRAW dataset. Models with different hyperparameter configurations were trained on the miRAW training set, and the corresponding best models were selected based on their performance on the miRAW validation set. Afterward, the selected models were then evaluated using the miRAW test set. At the start, the hyperparameters ${d}_{1}$, $ks$, and ${p}_{0}$ were initialized to 256, 9, and 0.5, respectively, and the hyperparameter ${n}_{h}$ was optimized in the first step. Table 3 presents the evaluation of models using $score$ values, and from these results, the optimal value for ${n}_{h}$ was determined to be 1. Subsequently, the parameters were adjusted sequentially, building upon the previous step. Ultimately, the best combination of hyperparameters was determined as ${n}_{h}=1, {d}_{1}=256, ks=9$, and ${p}_{0}=0.5$.

**Table 3:** Hyperparameter tuning experiments

	Accuracy (%)	Sensitivity (%)	Specificity (%)	PPV (%)	NPV (%)	F1 score (%)	Score
$${n}_{h}$$							
**1**	96.47	95.85	97.10	97.06	95.90	96.45	**5.7883**
2	94.89	94.47	95.30	95.25	94.53	94.86	5.6930
4	96.46	95.43	97.48	97.42	95.53	96.42	5.7874
8	93.74	94.71	92.78	92.90	94.62	93.80	5.6255
$${d}_{1}$$							
128	95.33	95.72	94.94	94.98	95.69	95.35	5.7201
**256**	96.47	95.85	97.10	97.06	95.90	96.45	**5.7883**
512	93.21	93.60	92.82	92.87	93.56	93.23	5.5929
1024	96.31	96.07	96.56	96.53	96.10	96.30	5.7787
$$ks$$							
1	87.47	93.26	81.69	83.57	92.39	88.15	5.2653
5	96.28	96.20	96.36	96.35	96.21	96.28	5.7768
**9**	96.47	95.85	97.10	97.06	95.90	96.45	**5.7883**
13	95.28	95.46	95.10	95.11	95.45	95.29	5.7169
$${p}_{0}$$							
0.25	93.13	94.97	91.29	91.59	94.78	93.25	5.5901
**0.5**	96.47	95.85	97.10	97.06	95.90	96.45	**5.7883**
0.75	95.91	94.79	97.03	96.96	94.91	95.86	5.7546
1.0	95.97	96.50	95.44	95.48	96.47	95.99	5.7585

The bold font indicates the best performance

### TEC-miTarget outperforms the state-of-the-art methods

TEC-miTarget was trained using the optimal hyperparameters and compared with the state-of-the-art methods. Specifically, TEC-miTarget was compared with miRAW, DeepMirTar, miTAR, and GraphTar at the sequence level, and compared with deepTarget, deepTargetPro, TargetNet, PITA, mirSVR, miRDB, microT, and Targetscan at the transcript level.

### TEC-miTarget outperforms the state-of-the-art methods at the sequence-level prediction

In this section, we evaluated the performance of TEC-miTarget at the sequence level, compared to miRAW, DeepMirTar, miTAR, and GraphTar. We first assessed the performance of the models on the miRAW dataset. Table 4 and Additional file 1: Figure S1A demonstrate the superior performance of TEC-miTarget across all evaluation metrics on the miRAW test set. Specifically, TEC-miTarget achieves the following percentage improvements for each metric: accuracy (+ 1.76%), sensitivity (+ 1.11%), specificity (+ 2.43%), PPV (+ 2.31%), NPV (+ 1.25%), and F1 score (+ 1.71%), compared to the best performance of the other four models. Moreover, as shown in Table 4 and Additional file 1: Figure S1B, TEC-miTarget surpasses the maximum values of the other four models on the miRAW independent test set across most metrics, including accuracy (+ 1.52%), specificity (+ 3.32%), NPV (+ 0.01%), and F1 score (+ 0.36%).

**Table 4:** Performance comparison between TEC-miTarget, miRAW, DeepMirTar, miTAR, and GraphTar on the miRAW dataset

	Accuracy (%)	Sensitivity (%)	Specificity (%)	PPV (%)	NPV (%)	F1 score (%)
*miRAW test set*						
miRAW	93.50	93.50	93.80	93.50	93.20	93.50
DeepMirTar	87.50	87.50	87.50	87.67	87.33	87.59
miTAR	93.94	93.94	93.94	94.03	93.85	93.98
GraphTar	94.80	94.80	94.80	94.87	94.72	94.83
TEC-miTarget	**96.47**	**95.85**	**97.10**	**97.06**	**95.90**	**96.45**
*miRAW independent test set*						
miRAW	91.30	93.10	36.30	**97.80**	15.00	95.40
DeepMirTar	86.54	86.55	86.53	87.02	86.04	86.78
miTAR	93.79	93.81	93.76	94.01	93.55	93.91
GraphTar	94.28	**94.26**	94.29	94.52	94.02	94.39
TEC-miTarget	**95.71**	94.08	**97.42**	97.44	**94.03**	**95.73**

The bold font indicates the best performance

Then, we compared the performance of the models on the DeepMirTar dataset. As shown in Table 5 and Additional file 1: Figure S1C, TEC-miTarget also demonstrates superior performance across all metrics. On the DeepMirTar test set, TEC-miTarget outperforms the best performance of the other four models in terms of accuracy (+ 4.21%), sensitivity (+ 3.95%), specificity (+ 3.86%), PPV (+ 3.99%), NPV (+ 4.09%), and F1 score (+ 4.19%). Moreover, on the DeepMirTar independent test set, TEC-miTarget showcases significant improvements in accuracy (+ 8.33%) and sensitivity (+ 8.33%). It is worth noting that the pre-trained word2vec model of GraphTar fails to encode some sequences in the DeepMirTar independent test set. Consequently, we had to label the corresponding samples as false negative pairs, which led to GraphTar exhibiting poor performance on the DeepMirTar independent test set.

**Table 5:** Performance comparison between TEC-miTarget, miRAW, DeepMirTar, miTAR, and GraphTar on the DeepMirTar dataset

	Accuracy (%)	Sensitivity (%)	Specificity (%)	PPV (%)	NPV (%)	F1 score (%)
*DeepMirTar test set*						
miRAW	90.22	90.25	90.20	90.25	90.18	90.25
DeepMirTar	93.48	92.35	94.79	94.64	92.56	93.48
miTAR	92.74	92.74	92.74	92.80	92.67	92.77
GraphTar	92.22	92.21	92.23	92.26	92.16	92.23
TEC-miTarget	**97.42**	**96.40**	**98.45**	**98.42**	**96.46**	**97.40**
*DeepMirTar independent test set*						
miRAW	40.63	40.63	NA	NA	NA	NA
DeepMirTar	50.00	50.00	NA	NA	NA	NA
miTAR	75.00	75.00	NA	NA	NA	NA
GraphTar	34.72	34.72	NA	NA	NA	NA
TEC-miTarget	**81.25**	**81.25**	NA	NA	NA	NA

The bold font indicates the best performance

NA represents that the value can’t be calculated because the corresponding dataset consists of only positive pairs

### TEC-miTarget outperforms the state-of-the-art methods at the transcript level prediction

TEC-miTarget was also evaluated at the "transcript level prediction". As shown in Table 6 and Additional file 1: Figure S2, TEC-miTarget consistently demonstrates stable and commendable performance across ten deepTargetPro test sets, highlighting the excellent generalization ability of TEC-miTarget.

**Table 6:** The performance of TEC-miTarget on ten deepTargetPro test sets

	Accuracy (%)	Sensitivity (%)	Specificity (%)	PPV (%)	NPV (%)	F1 score (%)
Test set 1	80.15	78.89	81.29	79.31	80.91	79.10
Test set 2	80.42	79.50	81.29	80.30	80.52	79.90
Test set 3	80.07	78.76	81.29	79.58	80.52	79.17
Test set 4	78.35	75.25	81.29	79.26	77.57	77.20
Test set 5	79.75	78.12	81.29	79.74	79.76	78.92
Test set 6	79.55	77.66	81.29	79.31	79.76	78.48
Test set 7	79.21	76.98	81.29	79.42	79.02	78.18
Test set 8	81.08	80.84	81.29	79.79	82.28	80.31
Test set 9	80.63	79.90	81.29	79.69	81.49	79.79
Test set 10	80.51	79.70	81.29	80.30	80.71	80.00
Average	79.97	78.56	81.29	79.67	80.25	79.11

We first compared TEC-miTarget’s performance with existing deep learning approaches, such as deepTarget, deepTargetPro, and TargetNet. The results, as shown in Table 7, illustrate that TEC-miTarget outperforms deepTargetPro across all evaluation metrics on ten test sets. Specifically, TEC-miTarget achieves the following percentage increases: accuracy (+ 1.93%), sensitivity (+ 3.05%), specificity (+ 0.91%), PPV (+ 1.50%), NPV (+ 2.33%), and F1 score (+ 2.30%), compared to deepTargetPro. Furthermore, TEC-miTarget significantly outperforms deepTarget in terms of accuracy, sensitivity, NPV, and F1 score, and surpasses TargetNet in terms of accuracy, specificity, PPV, and F1 score. Overall, TEC-miTarget demonstrates the best F1 score, which is a more important metric reflecting the comprehensive performance of the models. These results demonstrate the superior performance of TEC-miTarget.

**Table 7:** Average performance comparison between the deep learning approaches and TEC-miTarget on ten deepTargetPro test sets

	Accuracy (%)	Sensitivity (%)	Specificity (%)	PPV (%)	NPV (%)	F1 score (%)
deepTarget	65.21	34.77	**93.54**	**83.32**	60.64	49.04
deepTargetPro	78.04	75.51	80.38	78.17	77.92	76.81
TargetNet	72.61	**95.08**	51.67	64.69	**91.90**	76.99
TEC-miTarget	**79.97**	78.56	81.29	79.67	80.25	**79.11**

The bold font indicates the best performance

Then, we compare TEC-miTarget’s performance with other widely used seed-match-based methods (such as PITA, mirSVR, miRDB, microT, and Targetscan) in the past decades. As demonstrated in Table 8, TEC-miTarget stands out as the top performer. Specifically, TEC-miTarget significantly outperforms other models across various metrics including accuracy, sensitivity, PPV, NPV, and F1 score. In contrast, while miRBD excels in specificity, it falls short in terms of other performance metrics.

**Table 8:** Average performance comparison between the seed-match-based methods and TEC-miTarget on ten deepTargetPro test sets

	Accuracy (%)	Sensitivity (%)	Specificity (%)	PPV (%)	NPV (%)	F1 score (%)
PITA	50.53	13.65	87.41	51.96	50.31	21.62
mirSVR	50.01	27.76	72.26	49.97	50.01	35.68
miRBD	53.73	12.39	**95.07**	71.35	52.05	21.10
microT	61.13	58.94	63.32	61.62	60.70	60.24
Targetscan	55.77	39.45	72.08	58.52	54.36	47.12
TEC-miTarget	**79.97**	**78.56**	81.29	**79.67**	**80.25**	**79.11**

The bold font indicates the best performance

### Visualization of comparative experimental results

**Fig. 3:**
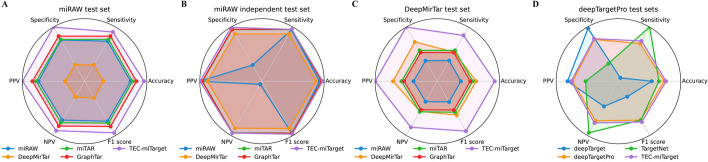
The radar charts of the comparative results. **A**–**C** The performance of TEC-miTarget on the miRAW test set (**A**), miRAW independent test set (**B**), and DeepMirTar test set (**C**), compared with miRAW, DeepMirTar, miTAR, and GraphTar. **D** The average performance of TEC-miTarget on deepTargetPro test sets, compared with deepTarget, deepTargetPro, and TargetNet

We also utilize the radar chart to visually show the comparative results on the miRAW test set, miRAW independent test set, DeepMirTar test set, and deepTargetPro test sets. As shown in Fig. 3, TEC-miTarget shows the largest square on the radar chart, providing further evidence of its accurate performance. In summary, TEC-miTarget surpasses state-of-the-art methods in both sequence-level and transcript-level prediction tasks, providing more accurate predictions for miRNA targets.

### TEC-miTarget reflects the binding region of miRNA CTS interaction

**Fig. 4:**
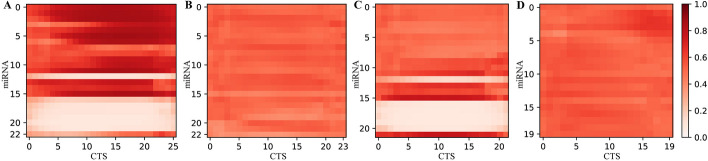
Base interaction probability maps. **A** TP pair. **B** TN pair. **C** FP pair. **D** FN pair

The base interaction probability map provides an intuitive representation of the interactions between a miRNA and its candidate target. As shown in Fig. 4, the predicted positive pairs (Fig. 4A, C) exhibit a stronger contrast in the base interaction probability map compared to predicted negative pairs (Fig. 4B, D). This contrast serves as the primary distinguishing feature between positive and negative predictions. Moreover, the average probabilities within the base interaction probability map for positive predictions tend to be higher than those for negative ones. Furthermore, the base interaction probability map demonstrates that the interactions between miRNA and its CTSs are primarily concentrated within the 5’ region of the miRNA. This observation underscores the precision of the features extracted by CNN.

### Ablation study about the real effectiveness of the encoder part

The encoder part of TEC-miTarget is composed of three sequential components: the base encoder (I), the positional encoder (II), and the transformer encoder (III). To assess the effectiveness of these components, ablation experiments were devised, considering that component I forms the fundamental module of the encoder part. Specifically, these experiments are structured as follows: (1) I +  II  +  III, (2) I  +  II, (3) I  +  III, and (4) I. Subsequently, we trained TEC-miTarget using the same training strategy and evaluated the performance of TEC-miTarget with each of the four different encoders serving as the encoder part of TEC-miTarget, respectively.

**Fig. 5:**
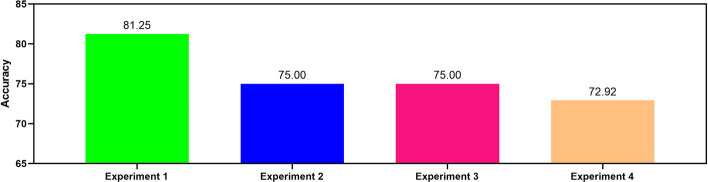
The performance of TEC-miTarget utilizing four different encoders. Models are trained on the DeepMirTar training set and evaluated on the DeepMirTar independent test set

As shown in Fig. 5, removing either the positional encoder (II) or the transformer encoder (III) from the encoder part results in a degradation of TEC-miTarget's performance (experiments 2 and 3). Specifically, the accuracy of TEC-miTarget decreases from 81.25% to 75.00%. Furthermore, the simultaneous removal of both the positional encoder (II) and the transformer encoder (III) leads to a more pronounced decrease in TEC-miTarget's performance, with the accuracy dropping to 72.92% (experiment 4). These results can be explained by analyzing the functions of the positional encoder and the transformer encoder. Primarily, the positional encoder integrates the positional information of RNA bases into the embeddings of RNA sequences, thereby enabling TEC-miTarget to comprehend the order or position of bases within RNA sequences. Moreover, the transformer encoder captures dependencies between RNA bases and generates rich contextualized representations for RNA sequences. These results demonstrate the effectiveness of the encoder part, underscoring the pivotal roles played by the positional encoder and the transformer encoder in enhancing TEC-miTarget's performance.

### Evaluation of TEC-miTarget’s execution speed

To assess the execution speed of TEC-miTarget, we measured the training time required for TEC-miTarget on the DeepMirTar training set, starting from the initiation of training until convergence. The experiments were conducted on an NVIDIA Tesla V100 with 32GB of memory, and the results were compared against other models. As shown in Table 9, TEC-miTarget achieved the second position in terms of training time, closely trailing the first-ranked DeepMirTar (27.66 min vs. 31.90 min). This is reasonable given that both TEC-miTarget and DeepMirTar have a higher parameter count, demanding more computational resources and consequently slowing down the training process. However, it's noteworthy that TEC-miTarget demonstrates effective performance during the testing phase. Table 9 illustrates the testing time for the models when assessed on the DeepMirTar test set, with TEC-miTarget as the second fastest approach. This is primarily due to our code optimization and the implementation of parallel inference methods. Overall, TEC-miTarget stands as a high-throughput method for miRNA target prediction.

**Table 9:** The execution speed evaluation

	Parameter count	Training time/min	Testing time/s
miRAW	471,951	17.91	2.85
DeepMirTar	29,203,396	31.90	4.94
miTAR	301,413	6.45	4.30
GraphTar	587,137	12.79	7.89
TEC-miTarget	26,691,717	27.66	4.01

## Discussion

Predicting miRNA targets plays a crucial role in understanding the significant functions of miRNAs in gene expression regulation. Over time, miRNA target prediction methods have witnessed remarkable advancements, transitioning from initial heuristic approaches to the current deep learning methods. However, the performance of existing approaches, including miRaw, DeepMirTar, and deepTargetPro, still requires further improvement to meet the demands of real-world applications. Consequently, there are growing demands to improve the performance of miRNA-target prediction models.

In the present study, we introduced a novel miRNA target prediction model called TEC-miTarget, which leverages the power of the transformer encoder and CNNs. Based on deep learning of ribonucleic acid sequences, the transformer encoder is employed to generate representations of miRNA and its CTS sequences. Afterward, these representations are fused into a three-dimensional array called the contact map containing the interaction information between miRNA and its CTSs. Additionally, taking cues from computer vision technologies, CNNs are utilized to extract features from the fused contact map. These extracted features enable the model to make accurate miRNA target site identification. Hyperparameters are fine-tuned through a series of hyperparameter tuning experiments, and subsequent comparative experiments are conducted using the final identified optimal hyperparameters. We first perform the comparative experiments at both the sequence level and transcript level for miRNA predictions. The results of comprehensive experiments demonstrate that TEC-miTarget consistently outperforms the three state-of-the-art models, including deep-learning-based and seed-match-based approaches. Furthermore, the base interaction probability map serves as an intuitive representation of the interactions between miRNA and its candidate target site, aiding in the interpretation of the model's predictions.

**Fig. 6:**
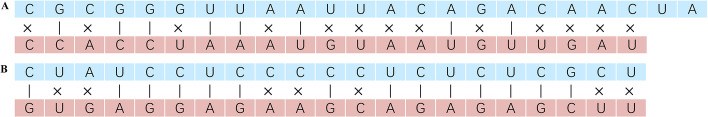
Waston Crick pairing for some atypical positive pair (**A**) and negative pair (**B**)

A significant challenge in miRNA target prediction at the transcript level lies in obtaining reliable candidate target sites (CTS) within a transcript [36]. Typically, the seed sequence, consisting of a few ribonucleotides located at the 5' end of the miRNA, is employed in combination with a specific matching strategy based on the Watson–Crick complementary condition [35] to select CTS within a transcript. However, these selection methods may introduce errors, leading to situations where certain transcripts have miRNA targets but cannot be detected out of any CTS using the selection methods. This phenomenon mainly occurs because the selection methods based on the seed sequence are one-sided or biased. As shown in Fig. 6, some positive pairs exhibit interactions that do not satisfy the selection methods due to a low ratio of paired bases (Fig. 6A). Conversely, in some negative pairs, the ratio of paired bases may be high (Fig. 6B). This discrepancy is attributed to the fact that miRNA-target interactions are not solely determined by the sequences but also depend on the structural characteristics of the miRNA and its target transcripts. Therefore, the base interactions determined solely by the Watson–Crick complementary condition may not accurately represent the true binding sites of miRNA and its targets. In this study, we employ the 13-mer-m9 method to identify CTS within transcripts, which yields superior performance compared to alternative strategies such as offset-9-mer-m7, as demonstrated in Additional file 1: Table S1. For more details on the offset-9-mer-m7, refer to deepTargetPro [23].

## Conclusions

Overall, our proposed TEC-miTarget model, utilizing natural language processing and computer vision technologies, surpasses other state-of-the-art methods in terms of several evaluation metrics through a series of comparative experiments. TEC-miTarget offers fresh insights into miRNA target prediction and represents a significant advancement in this field.

### Supplementary Information

**Additional file 1. Figure S1.** The distribution of predictions, the receiver operating characteristic, and precision-recall curves at sequence level evaluation. **Figure S2.** The distribution of predictions, the receiver operating characteristic, and precision-recall curves at transcript-level evaluation. **Table S1.** TEC-miTarget’s average performance using different selection strategies.

## Data Availability

TEC-miTarget is implemented in Python and is available on GitHub (https://github.com/tingpeng17/TEC-miTarget) aligned with the datasets used in this study. In addition, the model weights are available on Google Drive (https://drive.google.com/file/d/1L9eQYseXn1cctfl9jEHZ8Z_mpeA_vcKF/view?usp=drive_link).

## Abbreviations

RNA: Ribonucleic acid
miRNA: MicroRNA
NLP: Natural language processing
CNN: Convolutional neural network
CLIPL: Crosslinking and immunoprecipitation followed by RNA ligation
CLASH: Crosslinking, ligation, and sequencing of hybrids
SdAE: Stacked denoised autoencoder
CTS: Candidate target sites
RNN: Recurrent neural network
PPV: Positive predictive value
NPV: Negative predictive value
AUC: Area under the receiver operating characteristic
ROC: Receiver operating characteristic
AUPR: Area under the precision-recall curve
BCE: Binary cross entropy
