# Supplementary material for: TEC-miTarget: enhancing microRNA target prediction based on deep learning of ribonucleic acid sequences
Source: BMC Bioinformatics. 2024 Apr 20;25:159. doi: 10.1186/s12859-024-05780-z (PMC11032603; doi:10.1186/s12859-024-05780-z)
Supplement: Supplementary file 1 — Additional file 1. Figure S1. The distribution of predictions, the receiver operating characteristic, and precision-recall curves at sequence level evaluation. Figure S2. The distribution of predictions, the receiver operating characteristic, and precision-recall curves at transcript-level evaluation. Table S1. TEC-miTarget’s average performance using different selection strategies. [file 12859_2024_5780_MOESM1_ESM.docx]

# Supplementary Information


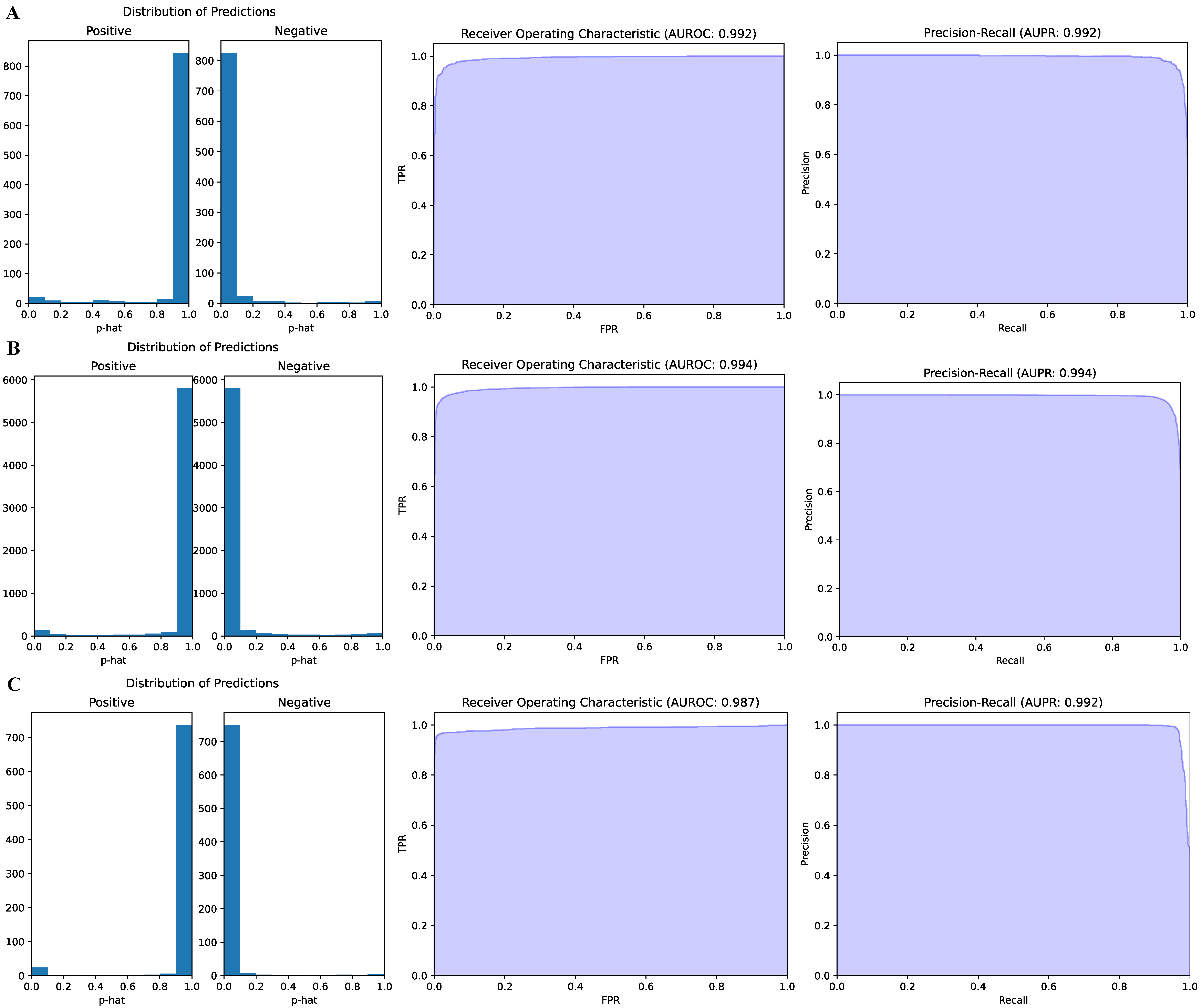


Figure S1. The distribution of predictions, the receiver operating characteristic, and precision-recall curves at sequence level evaluation. (A-B) TEC-miTarget is trained on the miRAW training set and evaluated on the miRAW test set (A) and miRAW independent test set (B). (C) TEC-miTarget is trained on the DeepMirTar training set and evaluated on the DeepMirTar test set.


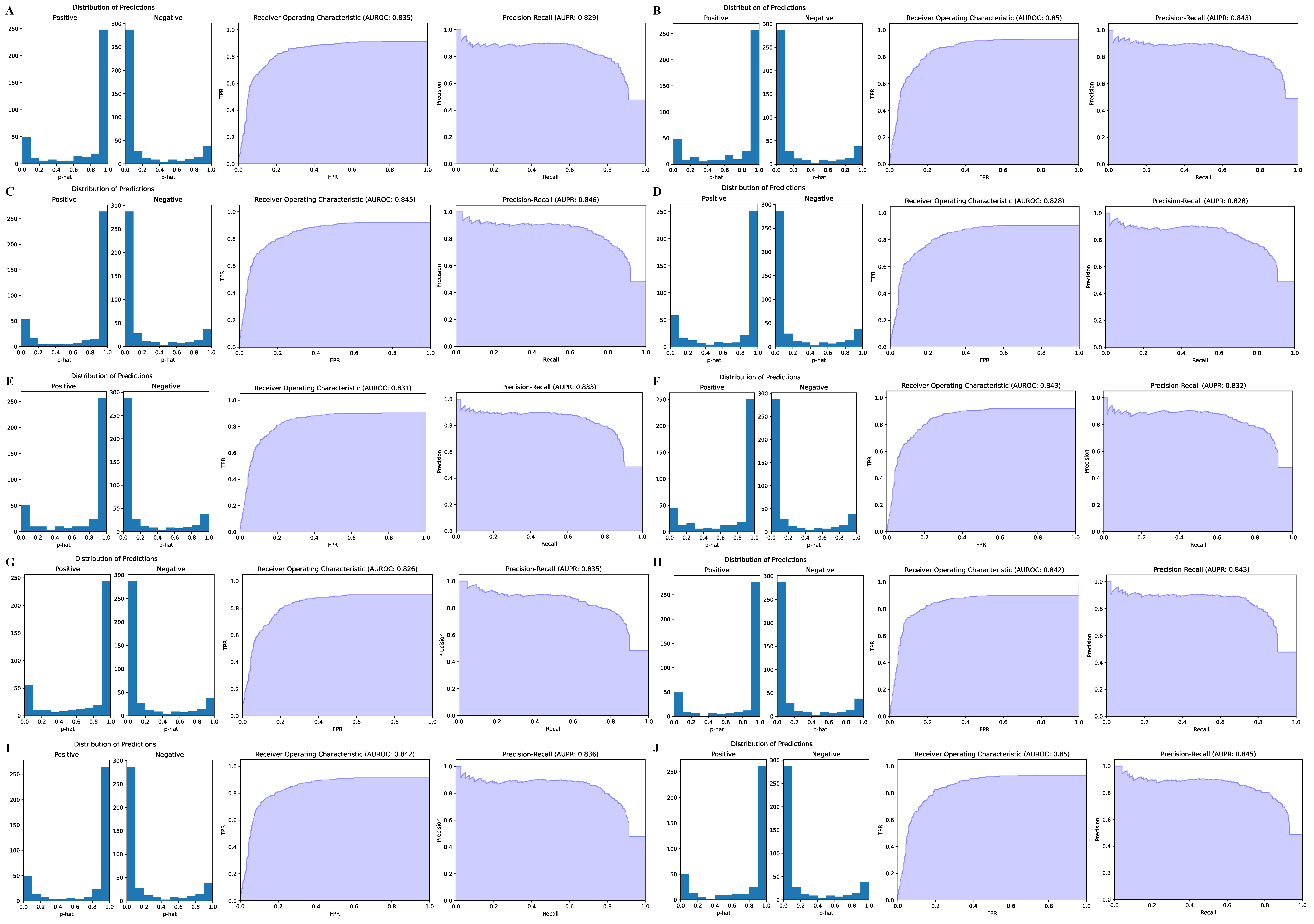


Figure S2. The distribution of predictions, the receiver operating characteristic, and precision-recall curves at transcript-level evaluation. TEC-miTarget is trained on the deepTargetPro training set and evaluated on the ten deepTargetPro test sets (A: test set 1, …, J: test set 10).

Table S1. TEC-miTarget’s average performance using different selection strategies

|  | Accuracy | Sensitivity | Specificity | PPV | NPV | F1 score | AUC | AUPR |
| --- | --- | --- | --- | --- | --- | --- | --- | --- |
| offset-9-mer-m7 | 79.39% | 73.24% | **85.13%** | **82.13%** | 77.33% | 77.42% | 0.8297 | 0.8083 |
| **13-mer-m9** | **79.97%** | **78.56%** | 81.29% | 79.67% | **80.25%** | **79.11%** | **0.8369** | **0.8393** |
